# Case Report: “Primary Immunodeficiency”—Severe Autoimmune Enteropathy in a Pediatric Heart Transplant Recipient Treated With Abatacept and Alemtuzumab

**DOI:** 10.3389/fimmu.2022.863218

**Published:** 2022-04-05

**Authors:** Elizaveta Kalaidina, Elizabeth C. Utterson, Deepa Mokshagundam, Mai He, Shalini Shenoy, Megan A. Cooper

**Affiliations:** ^1^ Department of Medicine, Division of Allergy/Immunology, Washington University in St. Louis, St. Louis, MO, United States; ^2^ Department of Pediatrics, Division of Gastroenterology/Nutrition, Washington University in St. Louis, St. Louis, MO, United States; ^3^ Department of Pediatrics, Division of Pediatric Cardiology, Washington University in St. Louis, St. Louis, MO, United States; ^4^ Department of Pathology/Immunology, Washington University in St. Louis, St. Louis, MO, United States; ^5^ Department of Pediatrics, Division of Pediatric Hematology/Oncology, Washington University in St. Louis, St. Louis, MO, United States; ^6^ Department of Pediatrics, Division of Rheumatology/Immunology, Washington University in St. Louis, St. Louis, MO, United States

**Keywords:** T-cell deficiency, enteropathy, heart transplant, immune modulatory therapy, cardiac transplant

## Abstract

Disorders of immune dysregulation following heart transplantation in children have been reported; however, the management of such disorders remains uncertain and challenging. In this case report, we describe a clinical course of a child with severe autoimmune enteropathy after a heart transplant in infancy and detail a treatment approach with abatacept and alemtuzumab. A 21-month-old girl with a medical history of congenital dilated cardiomyopathy and heart transplantation at 2 months was evaluated for chronic hematochezia. The patient underwent an extensive workup, including endoscopic biopsy which showed crypt apoptosis, similar to that seen with graft-versus-host disease (GVHD). Results of her immune workup were consistent with status post-thymectomy but also demonstrated evidence of immune dysregulation. Specifically, her immune phenotype at diagnosis demonstrated T-cell lymphopenia, restricted TCR repertoire and skewing of T-cell compartment toward memory phenotype, increase in serum soluble ILR2a, and hypergammaglobulinemia. In the absence of response to more standard immune modulation, the patient was treated with CTLA4-Ig (abatacept), followed by a combination of abatacept and a JAK inhibitor and, finally, a combination of abatacept and alemtuzumab. Following therapy with alemtuzumab, the patient achieved remission for the first time in her life. Her clinical course was complicated by a relapse after 6 months which again readily responded to alemtuzumab. Ultimately, despite these remissions, the patient suffered an additional relapse. This case highlights the challenges of neonatal thymectomy and adds new insights into the pathogenesis, diagnosis, and management of severe autoimmune enteropathy in pediatric heart transplant recipients.

## Introduction

Heart transplantation in infancy represents a complex challenge to the developing immune system. During the procedure, the thymus is frequently removed to allow for sternotomy and cannulation of the great vessels. It is now appreciated that thymectomy leaves a characteristic imprint on the immune system, similar to that observed in immunosenescence, and the impact of thymectomy performed in the neonatal period is even more profound (1–3). In addition, the use of immunosuppressive drugs required to prevent rejection, such as antithymocyte globulin and tacrolimus, further depletes the developing T-cell compartment present at the time of treatment. The interplay of these factors creates a unique immune phenotype in children who undergo cardiac transplantation in infancy, which has been characterized in a limited number of studies. Notably, despite peripheral proliferation of residual T cells and restoration of near-normal numbers of CD3^+^ T cells in the blood, the phenotype of such T cells is significantly altered compared with healthy children. In the absence of normal thymic output and negative selection in the thymus, children who have undergone thymectomy have restricted T-cell receptor (TCR) diversity and skewing of T-cell phenotype to memory (CD45RO^+^) cells, which can sometimes lead to impaired T-cell-dependent antibody response to pathogens and overproduction of antibodies to self-antigens (4, 5).

Increased risk of *de-novo* autoimmune disorders has been reported in recipients of solid organs and is generally thought to be related to immune dysregulation from immunosuppressive drugs. Marcus et al. reported the largest group of pediatric solid organ recipients to date, of which 103 patients were heart transplant recipients. In this study, autoimmune disorders, most commonly autoimmune cytopenias, inflammatory bowel disease (IBD), and IBD-like, were observed in pediatric heart recipients with a prevalence of 9.7% (3). It remains a question, however, whether immune-mediated disorders following heart transplantation share the same pathophysiology (and therefore amenable to the same treatments) with those following other solid organ transplantation, considering the unique immune phenotype of these patients. Management of refractory autoimmune disorders in pediatric heart transplant recipients is challenging. Here, we describe a clinical course of a child who was diagnosed with severe autoimmune enteropathy after a heart transplant in infancy and treated with a combination of abatacept and alemtuzumab.

## Case Description

A 21-month-old girl was evaluated for chronic diarrhea. Her medical history was significant for dilated cardiomyopathy associated with a pathogenic variant in *MYBPC3* for which she underwent ABO-compatible, complement-dependent cytotoxicity crossmatch negative orthotopic heart transplantation at the age of 2 months. Her diarrhea started approximately 6 months after transplantation as intermittent non-bloody loose stools, which gradually progressed to persistent hematochezia which required daily transfusions of red blood cells (RBCs). Due to her gastrointestinal (GI) illness and feeding intolerance, at 1 year of age, she was only at the 26th percentile for weight and 13th percentile for height and dependent on gastrostomy tube feedings. Her physical exam revealed a small child with no skin rash, lymphadenopathy, or organomegaly. Her thymic function prior to cardiac transplant and thymectomy is unknown, as routine neonatal screening for severe combined immunodeficiency (SCID) was not yet in place when she was born. At 5 months of age, she had a repeat newborn screen that demonstrated absent T-cell receptor excision circles (TRECs). Upon referral to the immunology consulting service, clinical assessment of her T-lymphocyte compartment at 21 months showed low recent thymic emigrants (CD4RTE, % of CD3^+^CD4^+^ that are CD45RA^+^CD31^+^) (**Table 1**). CD45RA/RO analysis of CD4 cells demonstrated a significant skewing to memory phenotype. TCRVβ spectratyping demonstrated an altered T-cell repertoire with 15 oligoclonal families. These findings are consistent with thymectomy including T-cell lymphopenia, T-cell repertoire restriction, and skewing of T-cell population to memory phenotype. Other pertinent findings included elevated serum IIL2Ra, indicative of a strong antigen stimulation of T cells, and hypergammaglobulinemia, thought to be due to overactivation of B cells in the absence of T-cell inhibitory signals. Genetic testing for known causes of SCID and combined immunodeficiencies was negative (clinical 207 gene panel). An extensive infectious workup was negative with the exception of persistent human herpesvirus 6 (HHV-6) viremia, which had been previously treated with ganciclovir but did not improve the diarrhea. Endoscopic biopsy of the patient’s colon showed crypt apoptosis, with a pathologic appearance of graft-versus-host disease (GVHD) after allogeneic hematopoietic stem cell transplantation (HSCT). A biopsy of her heart showed no evidence of graft rejection or GVHD.

**Table 1:** Immunologic evaluation of the patient between 5 and 36 months of age.

Age:	5 months	13 months	21 months	36 months	Ref range
TREC, cycle threshold (CT)	45 (absent)				<36
TREC, copies/10^6^ CD3^+^ T cells			Undetectable		>6,794
CD3, cells/μl		274	396	<55	2,100–6,200
CD4, cells/μl		64	122	<35	1,300–3,400
CD8, cells/μl		120	122	<45	620–2,000
CD19, cells/μl		592	822	<25	720–2,600
CD56/16, cells/μl		1,065	183	>2,000	180–920
CD4RTE, % of CD4^+^ T cells			0.8		25.8–68
CD45RA, % of CD4^+^ T cells			2		63–91
CD45RO, % of CD4^+^ T cells			98		7–20
TCRVβ repertoire			Predominantly oligoclonal and polyclonal non-Gaussian		Polyclonal Gaussian
IgG, mg/dl			1,833		345–1,213
Soluble IL2Ra, IU/ml			1,280	1,382	45–1,105

CD4RTE, CD4 T-cell recent thymic emigrants (percentage of CD3+CD4+ cells that are CD31+ CD45RA+cells).

The patient’s immune phenotype was consistent with a history of thymectomy due to cardiac transplant, but was also similar to that seen in Omenn syndrome, an immune dysregulation syndrome in patients with “leaky” SCID characterized by the presence of autoreactive oligoclonal T cells (6). In Omenn syndrome, autoreactive T cells infiltrate the skin, GI tract, and other organs leading to erythroderma, chronic diarrhea, and failure to thrive (7). A similar phenomenon of autoreactive T cells has been recognized in some autologous HSCT recipients (8, 9). Theoretically, patients undergoing autologous HCST should be exempt from GVHD, since there is no HLA disparity between infused stem cells and the recipient (9). Interestingly, pathology similar to allogeneic GVHD is present in ~10% of autologous HCST recipients, termed “autologous GVHD” or “auto-GVHD,” but is typically mild and limited to the skin (8). However, a more severe presentation with gut involvement has been described, including patients with persistent diarrhea and GVHD-like pathology (9). Based on the immune phenotype and gastrointestinal tract pathology in our patient and the previously described syndromes, we hypothesized that the patient’s enteropathy represented autologous GVHD enteropathy due to T-cell dysregulation.

**Figure 1:**
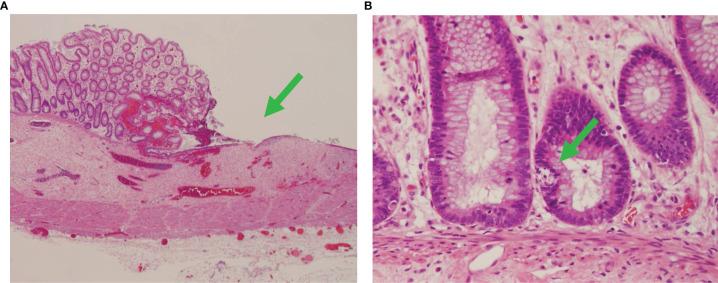
Autologous graft-versus-host disease in the colon. Fragments of colon tissue from the patient demonstrating diffuse subtotal loss of mucosa with marked epithelial apoptosis (green arrows) and intermittent ulcers, consistent with severe autologous graft-versus-host disease. **(A)** ×40 and **(B)** ×400 magnification.

**Figure 2:**
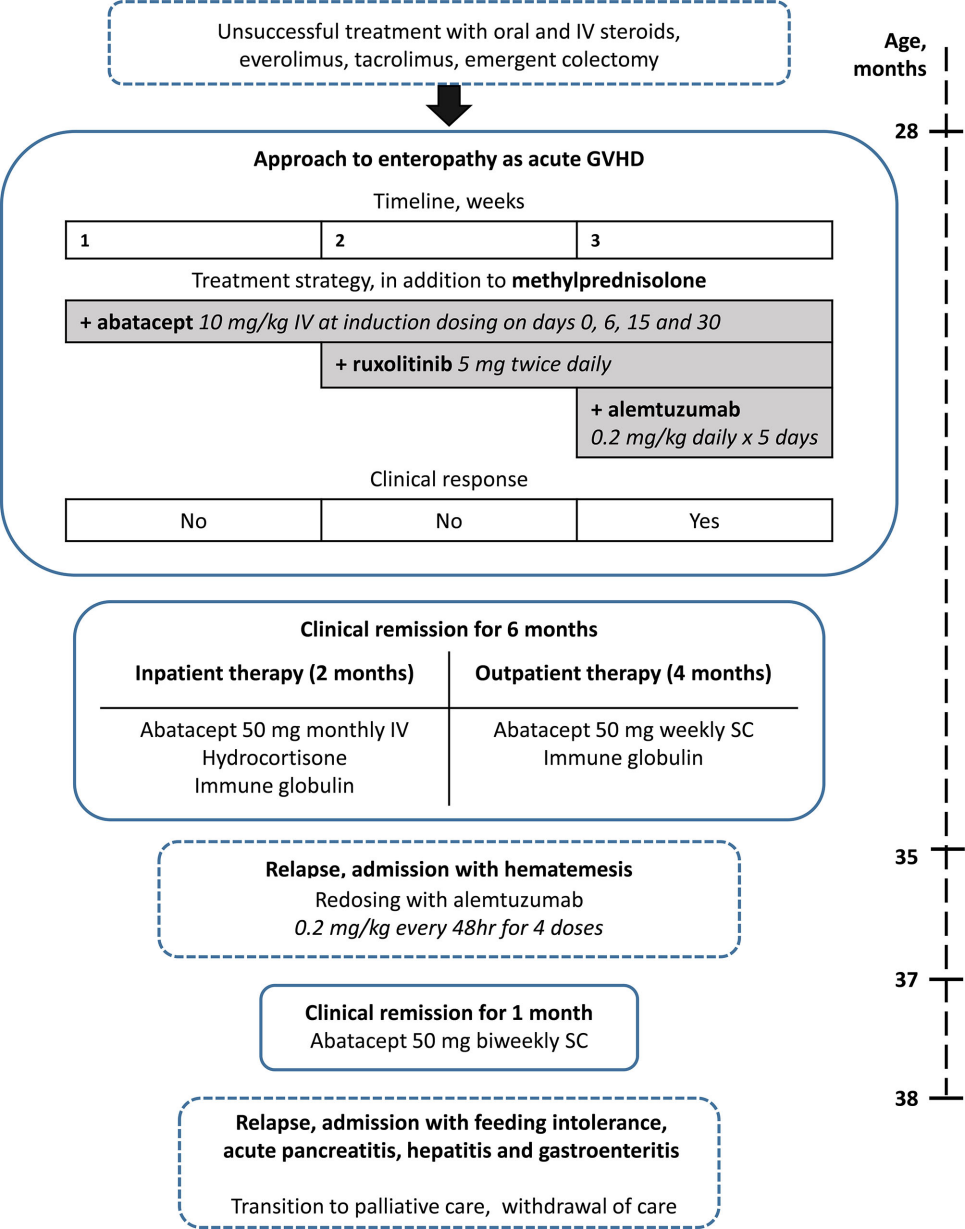
Treatment of enteropathy. The table displays the time course, details, and outcomes of treatment of enteropathy from 28 to 38 months.

Her enteropathy was initially treated with methylprednisolone; however, she continued to have GI bleeding despite this therapy. In an effort to promote regulatory T-cell function and suppress autoreactive effector T cells, her initial immunosuppressive therapy with tacrolimus was changed to everolimus, an mTOR inhibitor. However, she continued to have recurrent gastrointestinal bleeding, ultimately requiring a subtotal colectomy, proctectomy, and end ileostomy. A biopsy taken from the large bowel at the time of the laparotomy again demonstrated features of acute GVHD, consistent with T-cell-mediated enteropathy (**Figure 1**). Despite continued treatment with everolimus and steroids, GI bleeding from ulceration in the small intestine persisted. We therefore approached treatment similar to that of patients with allogeneic GVHD and initiated abatacept therapy (10 mg/kg at induction dosing interval on days 0, 6, 15, and 30). This was quickly followed by a combination of abatacept with ruxolitinib (5 mg twice daily) and, finally, a combination of abatacept with alemtuzumab (0.2 mg/kg daily × 5 days) (**Figure 2**). Following alemtuzumab, she had a clinical response with cessation of gastrointestinal bleeding accompanied by normalization of the level of soluble IL2Ra (920 IU/ml). Abatacept was continued as maintenance therapy at 50 mg/week subcutaneously. In addition, she received weekly subcutaneous immunoglobulin infusions for secondary hypogammaglobinemia. The patient was discharged after 160 days in the hospital in stable condition on weekly abatacept, antimicrobial prophylaxis, and weekly subcutaneous immunoglobulin.

After almost 4 months of sustained remission on outpatient therapy, which was the longest period she was not hospitalized in her life, abatacept was spaced out to every 14 days. Unfortunately, she developed hematemesis and was readmitted to the hospital. Endoscopic biopsy of the ileum revealed crypt apoptosis and gland dropout with minimal inflammation, similar to changes observed at the time of her laparoscopy and consistent with acute GVHD-like pathology. Her soluble IL2Ra increased to 1,382 IU/ml, indicating T-cell activation. Other causes of GI bleeding were excluded. At this point, 8 months post-treatment with alemtuzumab, her peripheral blood flow cytometry revealed undetectable levels of T and B cells, indicating that her peripheral T- and B-cell compartments did not reconstitute. Her NK cells, by contrast, had recovered and were increased to more than 2,000 cells/μl. We hypothesized that the recurrence of GI bleeding was caused by autoreactive T cells already recruited to the site of self-antigen presentation in the small intestine. Therefore, pulse therapy with alemtuzumab was reinstated at 0.2 mg/kg every 48 h for 4 doses which again resulted in the resolution of GI bleeding. The patient was discharged on maintenance therapy with biweekly abatacept. However, within a month after discharge, she represented to the hospital with feeding intolerance and was diagnosed with acute pancreatitis, hepatitis, and gastroenteritis. The option of allogeneic HCST was discussed, but deemed very high risk, considering the patient’s clinical status, her history of cardiac transplant, and the uncertainty of T-cell reconstitution with athymia. Thymic tissue transplantation was also discussed, but is not currently clinically available for this indication. Despite an extensive workup, the cause of her acute worsening remained uncertain but was thought to be multifactorial, including possible HHV-6 infection and drug-induced toxicity. Her condition progressively deteriorated as she developed pancytopenia, ulcerations at the ostomy site, and GI bleeding. At that point, the decision was made to transition her care to comfort measures, and the patient succumbed to her illness.

## Discussion

Autoimmune enteropathy has been described as a component of inborn errors of immunity such as immunodysregulation polyendocrinopathy enteropathy X-linked (IPEX) and other disorders with loss of T-cell tolerance, as well as in association with other non-syndromic immune-mediated conditions and as an isolated disorder (10). Autoimmune enteropathy is a well-recognized complication of GVHD in patients following allogeneic HSCT; however, recipients of autologous transplants can also have auto-GVHD and autoimmune enteropathy (8, 9). Recent evidence suggests that in comparison to allogeneic GVHD driven by the disparity in donor and recipient HLA, the mechanism underlying autologous GVHD may be due to impaired reconstitution of the thymic medulla resulting in the loss of T-cell tolerance (11, 12).

Treatment of steroid-refractory cases of autoimmune enteropathy can be challenging, and multiple immune modulatory drugs have been tried with varied success. When refractory to medical therapies, HCST has been used to reset the immune system (13, 14). Management of immune dysregulation in transplant patients is further complicated due to the inherent requirement for immunosuppressive medications. Reports on autoimmune enteropathy and its management and outcomes in pediatric heart transplant recipients are scarce (15). Our treatment approach to this case was based on the findings of acute GVHD-like pathology and leaky SCID-like immune phenotype of the patient. We observed a favorable and reproducible clinical response to a combination of alemtuzumab and abatacept. The prompt clinical response to this immune modulatory therapy substantiated our hypothesis that immune dysregulation was indeed an underlying pathophysiology of enteropathy. It remains unclear why the response to abatacept and alemtuzumab did not translate into sustained remission. It is less likely that changing the abatacept dose to every 2 weeks precipitated the worsening of autoimmune enteropathy, considering its half-life of approximately 14 days. We speculate that the initial success with abatacept and alemtuzumab was counteracted by complications associated with secondary immune deficiency and a relapsing nature of the pathology.

This case illustrates the impact of neonatal heart transplantation and thymectomy on the developing immune system, alerts the physicians to consider autoimmunity as a potential cause of enteropathy in pediatric heart transplant recipients, and describes the approach to the management of severe autoimmune enteropathy with abatacept and alemtuzumab.

## Data Availability Statement

The original contributions presented in the study are included in the article/supplementary material. Further inquiries can be directed to the corresponding author.

## Ethics Statement

The studies involving human participants were reviewed and approved by the Washington University IRB. Written informed consent to participate in this study was provided by the participant’s legal guardian/next of kin.

## Author Contributions

EK and MC provided clinical care and wrote and edited the manuscript. EU, DM, MH, and SS provided clinical expertise and reviewed the manuscript. All authors contributed to the article and approved the submitted version.

## Funding

This research was supported by the Jeffrey Modell Diagnostic and Research Center for Primary Immunodeficiencies at St. Louis Children’s Hospital and the Center for Pediatric Immunology at St. Louis Children’s Hospital and Washington University Department of Pediatrics.

## Conflict of Interest

MC has received consulting fees from Enzyvant. SS reports a one-time service as a consultant for Bristol Myers Squibb advisory board meeting.

The remaining authors declare that the research was conducted in the absence of any commercial or financial relationships that could be construed as a potential conflict of interest.

## Publisher’s Note

All claims expressed in this article are solely those of the authors and do not necessarily represent those of their affiliated organizations, or those of the publisher, the editors and the reviewers. Any product that may be evaluated in this article, or claim that may be made by its manufacturer, is not guaranteed or endorsed by the publisher.
